# Cell-Mediated Mechanical Stability Enhancement of Biomimetic Collagen-Alginate Hydrogels: A Mechanistic Study on the Two-Dimensional Extracellular Matrix–Cell Interaction

**DOI:** 10.1021/acs.chemmater.5c03410

**Published:** 2026-04-06

**Authors:** Shuhan Feng, Sami Hietala, Juan José Valle-Delgado, Marko Vehkamäki, Alexandra Correia, Shiqi Wang

**Affiliations:** 1 Drug Research Program, Divisions of Faculty of Pharmacy, 124454 University of Helsinki, Helsinki FI-00014, Finland; 2 Institute of Biotechnology, Helsinki Institute of Life Science (HiLIFE), 124454 University of Helsinki, Helsinki FI-00014, Finland; 3 Department of Chemistry, Faculty of Science, 124454 University of Helsinki, Helsinki FI-00014, Finland; 4 Department of Bioproducts and Biosystems, School of Chemical Engineering, 174277 Aalto University, Aalto FI-00076, Finland

## Abstract

Cell–matrix interactions are a central topic in the field of biomimetic material mechanics. While the influence of matrix stiffness on cellular adhesion, spreading, and differentiation has been extensively investigated, the reciprocal impact of cells on the mechanical properties of biomimetic matrices remains less explored. In this work, we demonstrate that fibroblasts can remodel the mechanical properties of collagen-alginate hybrid hydrogel (CAH) matrices in a 2D culture. We found that, in the absence of cells, CAHs showed a progressive stiffness decline in the cell culture medium due to calcium ion release. In contrast, when fibroblasts were present, the stiffness of the hydrogels remained stable despite calcium ion release. This stabilization was collectively contributed by fibroblast activity and calcium deposition, with cells serving as mineral nucleation sites and reinforcing the local collagen network. Together, these results highlight the role of cells in reshaping the biomaterials’ mechanical properties and advance our understanding of the dynamic, reciprocal nature of cell–extracellular matrix interactions.

## Graphical abstract

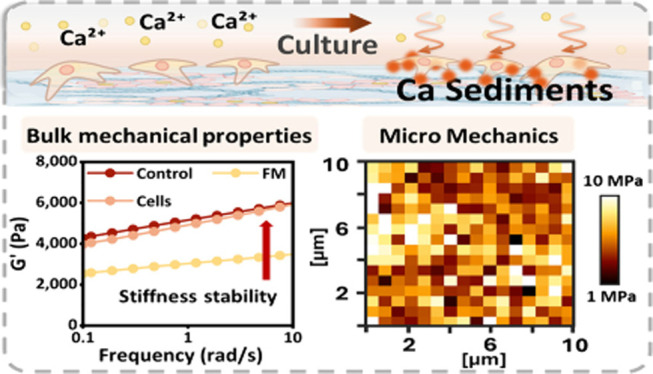

## 1 Introduction

The natural extracellular matrix (ECM) constitutes the cellular microenvironment, providing mechanical support, as well as essential biochemical and biophysical cues for cell survival. Cells connect with the ECM via their surface integrins and growth factor receptors, which sense and integrate various extracellular stimuli (e.g., mechanical, nutritional, and biochemical signals) and transduce them into intracellular signaling cascades, ultimately regulating gene expression and cellular phenotype.^1^ Therefore, the physicochemical properties of the ECM affect fundamental cellular behaviors, including proliferation, adhesion, migration, and differentiation.^2^ In turn, cellular differentiation and migration direct the organization of cells into distinct tissues, accompanied by the dynamic remodeling of the ECM into tissue-specific three-dimensional (3D) microenvironment. During continuous remodeling, cells secrete matrix metalloproteinases that degrade ECM components (e.g., collagen, fibronectin, and laminin) while simultaneously synthesizing new ECM to supplement the degraded matrix and sustain cellular activity.^3^ These dynamic cell-ECM interactions, often termed dynamic reciprocity or bidirectional crosstalk, endow tissues with a flexible and adaptive physical microenvironment.^1^

Since investigating cell-ECM interactions *in vivo* remains challenging, various hydrogels have been developed to mimic key features of natural ECM and provide controllable platforms for studying these interactions *in vitro*.^4^ These hydrogels are networks formed from biocompatible polymer precursors, exhibiting excellent hydrophilicity, swelling capacity, and water retention under physiological conditions.^5^ By incorporating functional peptide motifs (e.g., RGD, IKVAV, GRGDS), hydrogels provide cell-adhesive cues that regulate the coupling between the cytoskeleton and the hydrogel matrix.^6,7^ Furthermore, the mechanical properties of the hydrogel (e.g., stiffness, stress relaxation, creep, hysteresis) can be modulated by changing polymer composition or cross-linking strategies. The combination of essential biochemical and mechanical cues from hydrogel matrices effectively mimics the extracellular environment and supports long-term cellular functions.^8^

Recent studies have identified stiffness as a key determinant of the cellular response in hydrogel-based ECM systems. Variations in matrix bulk stiffness have been shown to modulate cell growth, proliferation, migration, and differentiation. For example, cells cultured on stiffer hydrogels typically exhibit a more spread morphology, enhanced motility, and increased mineral deposition compared with those cultured in soft environments.^9,10^ Mechanistically, a stiffer extracellular matrix enhances integrin clustering and focal adhesion maturation, strengthening actomyosin contractility. This mechanical feedback not only activates focal adhesion kinase (FAK) and downstream RhoA/ROCK signaling, but also triggers downstream pathways (e.g., MAPK/ERK, PI3K/Akt, and YAP/TAZ).^11^ These signaling cascades collectively reinforce cytoskeletal tension, promote cell spreading and migration, and ultimately regulate cell proliferation and lineage specification.^12^ Despite extensive studies on how matrix stiffness regulates cell behavior, the reciprocal effect of cell behavior on the mechanical properties of hydrogel matrices has often been underestimated. This is partially due to the fact that the cell-mediated ECM remodeling is a local effect at the single-cell level.^13^ Unless in the case of significant matrix degradation over long culture periods, the subtle stiffness variation induced by cells in the heterogeneous hydrogel matrix is barely detectable via bulk rheological methods.^14^

In this study, we sought to investigate cell–matrix interactions using collagen-alginate hydrogels (CAHs) under 2D culture conditions. As a classic ECM protein, collagen was incorporated to provide sufficient cell-adhesive sites within the hydrogel matrix.^15^ Alginate was introduced to enable mechanical modulation via Ca^2+^-mediated ionic cross-linking.^16^ By varying calcium concentrations (2, 10, 20, and 50 mM), we established a series of CAHs matrices with progressively increasing stiffness. Unexpectedly, the matrices exhibited a pronounced, cell density-dependent counterbalancing effect on mechanical losses caused by calcium ion release during culture. The stiffness-reinforcing effect was not limited to the pericellular microscale but was also detectable on a rotational rheometer. Motivated by these observations, we carefully investigated the stiffness-reinforcing mechanisms using scanning electron microscopy (SEM), energy-dispersive X-ray spectroscopy (EDS), and atomic force microscopy (AFM). Our results suggest that cell-mediated calcium deposition at the hydrogel-cell interface may represent an overlooked mechanism contributing to matrix stiffening. Considering the wide applications of alginate- and collagen-based hydrogels in tissue engineering and mechanobiology, our findings highlight the underappreciated role of calcium deposition and emphasize that these hydrogel substrates are not mechanically static but are actively shaped by cellular activity.

## 2 Experimental Section

### 2.1 Materials

Medium-viscosity sodium alginate (PRONOVA UP MVG, NovaMatrix; molecular weight > 200 kDa, guluronate content ≥ 60%) was purchased from Sigma-Aldrich. Type I collagen (calf skin) was obtained from Elastin Products Company, Inc. 5-Norbornene-2-methylamine (Norb-NH_2_) and tetrazine-amine monohydrochloride (Tz-NH_2_) were sourced from Tokyo Chemical Industry Co., Ltd. and MedChemExpress, respectively. Unless otherwise stated, all other chemicals were analytical grade, acquired from Sigma-Aldrich (UK), and used without further purification.

Dulbecco’s modified Eagle medium (DMEM; high glucose, containing l-glutamine), trypsin solution (2.5%, 10× in HBSS, without calcium and magnesium), nonessential amino acids (NEAA, 100×), and phosphate-buffered saline (PBS, 10×, without calcium and magnesium) were purchased from HyClone (Cytiva, Marlborough, MA, USA). Penicillin-streptomycin solution (10,000 U/mL) and heat-inactivated fetal bovine serum (FBS) were obtained from Thermo Fisher Scientific (Waltham, MA, USA). NIH 3T3 mouse embryonic fibroblasts were acquired from the American Type Culture Collection (ATCC, Manassas, VA, USA).

### 2.2 Synthesis of Collagen Methacrylamide (CMA)

To facilitate cell detachment, CMA possessing temperature-dependent reversible self-assembly properties, was synthesized based on the method of Drzewiecki et al.^17^ with some modifications. Briefly, the carboxyl groups of methacrylic acid were activated at 37 °C using 0.2 M 1-ethyl-3-(3-(dimethylamino)­propyl) carbodiimide (EDC) and 0.2 M *N*-hydroxysuccinimide (NHS) in 0.1 M MES buffer for 10 min. The activated methacrylic acid reacted with a collagen solution (2 mg/mL in 0.02 M acetic acid) at 4 °C for 22 h, forming CMA. The resulting CMA was dialyzed against 0.02 M acetic acid and lyophilized for storage and further use. The synthesized CMA forms a gel at 37 °C and transitions to liquid at 4 °C (Figure 1a).

**Figure 1:**
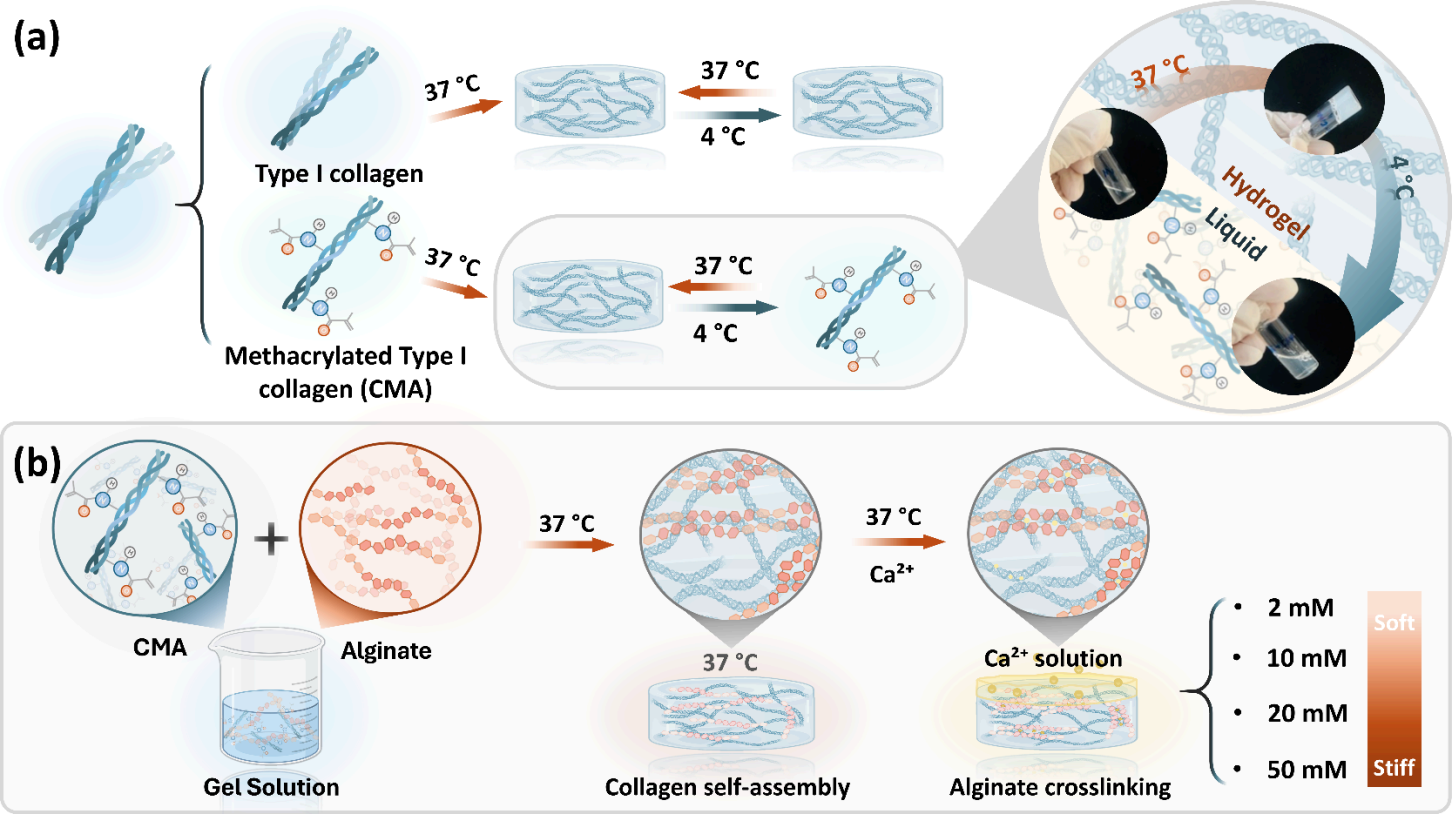
Schematic illustration of the preparation of CAHs. (a) Modification of collagen through methacrylation imparts thermosensitive properties. Inset images demonstrate the methacrylated collagen hydrogel transitions from gel at 37 °C to a liquid state at 4 °C. (b) Preparation of CAHs with tunable stiffness. Increasing gel stiffness is achieved by adjusting Ca^2+^ concentrations from 2 to 50 mM (2, 10, 20, and 50 mM).

### 2.3 Preparation of Tz- and Norb-Functionalized Alginate

Activated alginate was prepared by dissolving alginate (1 wt %) in MES buffer (0.1 M MES, 0.3 M NaCl, pH 6.5) and treating it with EDC/NHS (5-fold molar excess) at 37 °C for 10 min. The activated polymer was subsequently reacted with either Tz-NH_2_ or Norb-NH_2_ (0.2 equiv) under continuous stirring for 20 h at room temperature. Residual NHS esters were quenched using NH_2_OH·HCl, and both Tz- and Norb-functionalized alginate derivatives were purified by dialysis by decreasing NaCl concentrations.

^1^H NMR spectra of unmodified and functionalized (Norb and Tz) alginates were recorded on a Bruker Avance NEO 400 MHz spectrometer (Figure S1a). Lyophilized samples were dissolved in D_2_O (2.5 mg/mL), and the spectra were processed using MestReNova software. The degree of substitution of Norb groups (DS_Nb_) was determined based on the area integrals of the characteristic peaks at δ = 5.92, 6.08, and 6.18 ppm (2H, vinyl protons of the norbornene ring) relative to that at δ = 3.51–4.25 ppm (5H, alginate backbone), while the DS of Tz groups (DS_Tz_) was calculated from the area integrals of the characteristic peaks at δ = 10.33, 8.42, and 7.58 ppm (5H, aromatic protons of the tetrazine moiety) relative to that at δ = 3.49–4.22 ppm (5H, alginate backbone). FTIR spectra of unmodified and functionalized alginate were collected on a Thermo Scientific Nicolet iS50 FT-IR spectrometer equipped with an ATR accessory in the range 4000–400 cm^–1^ at room temperature (Figure S1b).

### 2.4 Fabrication of CAHs

CAHs were fabricated under sterile conditions following a two-step procedure: collagen self-assembly and alginate ionic cross-linking (Figure 1b). Initially, CMA and alginate were separately dissolved at room temperature in 0.02 M acetic acid and serum-free DMEM, respectively. CMA solution was combined with 10× DMEM (1:10 ratio), followed by addition of the alginate solution, adjusting the mixture to neutral pH (∼7.0) using 0.15 M NaOH. Final concentrations of CMA (2.5 mg/mL) and alginate (0.75% w/v) were achieved by dilution in serum-free DMEM. The resulting CAH precursor solution was transferred into disk-shaped molds and incubated at 37 °C for 4–5 h to allow collagen self-assembly. Subsequently, a series of calcium cross-linking solutions composed of serum-free DMEM supplemented with different concentrations of Ca^2+^ (2, 10, 25, and 50 mM) was added, and the hydrogels were incubated at 37 °C for at least 12 h to allow complete alginate gelation. The resulting gels were defined as fresh, untreated CAHs (2, 10, 25, and 50 mM).

### 2.5 In Vitro Cell Culture and Assessments

Fibroblast culture medium consisted of high-glucose DMEM supplemented with 10% fetal bovine serum (FBS), 1% penicillin-streptomycin, 1% HEPES, and 1% l-glutamine. NIH 3T3 fibroblasts were maintained in this medium at 37 °C in a humidified incubator with 5% CO_2_. The medium was replaced twice weekly, and cells were passaged at ∼90% confluency using 0.25% trypsin-EDTA. For cell culture, Ca^2+^-treated CAHs were washed twice with high-glucose DMEM prior to seeding NIH 3T3 cells onto their surfaces, followed by incubation for up to 4 days. To minimally preserve the relative structural stability of CAHs during culture, we supplemented the fibroblast culture medium with 2 mM Ca^2+^ when evaluating gel-related properties. This Ca^2+^ content was adopted with reference to the method of Kuo and Ma.^18^ Hereafter, this Ca^2+^-supplemented medium is termed fibroblast medium (FM).

Cell viability was assessed with a Live/Dead viability/cytotoxicity Kit (calcein-AM and ethidium homodimer-1, Thermo Fisher Scientific), followed by fluorescence imaging (EVOS FLoid Cell Imaging Station, Thermo Fisher Scientific) and flow cytometry (BD Accuri C6 Plus, BD Biosciences). The flow cytometry data were processed by FlowJo software (version 10.10, Becton Dickinson & Company, Ashland, OR, USA), and the live cell ratio was quantified.

Cell morphology was characterized using a Zeiss Celldiscoverer 7 system equipped with an LSM 900 Airyscan 2 confocal module. CAHs were prepared in cylindrical molds (diameter = 8 mm and height = 1 mm) prior to cell seeding. After 4 days of culture, samples were fixed with 4% paraformaldehyde, permeabilized with 0.5% Triton X-100, and stained with phalloidin-iFluor 594 and DAPI to visualize actin filaments and nuclei, respectively, before confocal imaging. Cell spreading area and aspect ratio were quantified from fluorescence micrographs by using Fiji (ImageJ, version 2.14.0). Images were spatially calibrated by using embedded microscope metadata prior to analysis. Cells were segmented by intensity-based thresholding with watershed separation followed by size and shape filtering to exclude debris and cell aggregates. The projected cell area was obtained from the segmented masks, and the aspect ratio was defined as the ratio of the major to minor axes of the fitted ellipse. At least 30 nonoverlapping fields of view from three independent samples were analyzed for each condition, with no fewer than 50 cells quantified per group.

### 2.6 Mechanical Characterization under Different Culture Conditions

CAH disks (height = 1 mm, diameter = 22 mm) were fabricated in 12-well plates. After gelation, the hydrogels were washed twice with FM to remove excess calcium ions. Samples were then incubated at 37 °C in PBS or FM for 1 or 4 days. For the cell-treated group, NIH 3T3 fibroblasts (1 × 10^4^ cells/well) were seeded onto CAH scaffolds. Samples were labeled PBS-1/4 day, FM-1/4 day, and Cell-1/4 day, respectively. After 1 or 4 days of incubation, hydrogel disks were placed between 20 mm parallel plates of a plate–plate rheometer (AR 2000, TA Instruments, New Castle, DE, USA). A frequency sweep (0.1–300 Hz at 1% strain) was conducted to assess the storage modulus (*G*′) within the linear viscoelastic region (Figure S2). All measurements were performed at physiological temperature (37 °C).

In addition, the 20mM CAH disks with 0, 10^2^, 10^3^, and 10^4^ cells as well as the front (medium interface) and back (well–plate interface) surfaces of 50 mM CAH disks seeded with different cell densities (0, 10^2^, and 10^4^ cells) were cultured in FM for 4 days prior to rheological testing to evaluate cell density-dependent effects. As a covalent cross-linking control, Tz- and Norb-modified alginate were mixed with collagen to form CAHs of identical formulation but without ionic cross-linking, seeded with 0, 10^2^, 10^3^, or 10^4^ cells, and cultured for 4 days before rheological measurements. All rheological measurements were performed under the same conditions as those described above.

Compression tests were performed with a texture analyzer (TA.XTPlus, Stable Micro Systems Ltd., UK) at room temperature using a radiused cylinder probe (P/0.5), compressing samples to a 30% initial height at 1 mm/s. The compressive modulus was defined as the slope of the stress–strain curve within the initial 30% strain (*n* = 4), which is commonly employed as an approximation of the Young’s modulus in soft hydrogel systems. Similar approaches have been applied in hydrogel mechanics research, such as using the linear region of the stress–strain curve in compression tests to determine Young’s modulus.^19^

Microscale surface mechanical mapping of CAHs was conducted by using a commercial atomic force microscope (NanoWizard IV XP BioScience AFM, JPK-Bruker, Berlin, Germany). Silicon nitride, V-shaped MLCT probes (D cantilevers) (Bruker, Camarillo, CA) were used to identify the hydrogels and quantify local stiffness (Young’s modulus). The probes were calibrated by measuring the deflection sensitivity against the bottom of a clean Petri dish, and the spring constant values (about 0.045 N/m) were obtained with the thermal tune method.^20^ The experiments were carried out in DMEM, and the temperature was controlled with a PetriDishHeater (JPK-Bruker; set temperature 37 °C; measured temperature in the center of the Petri dish, about 30 °C). Force mapping was applied in at least 5 different positions of the hydrogels, and in each position, indentation curves were collected in a 16 × 16-point array within 10 × 10 μm^2^ square areas. The indentations were conducted at 2 μm/s speed, with a maximum applied force of 2 nN. Young’s modulus values were obtained by analyzing the indentation curves with JPK Data Processing software (version 7.0.186, JPK-Bruker), applying the Hertz/Sneddon model and assuming a Poisson’s ratio of 0.3 and a tip half angle to edge of 17.5°.

### 2.7 Characterization of Free Calcium and Calcium Deposition

The released calcium from CAHs was quantified using a colorimetric calcium assay kit (ab102505, Abcam) following the manufacturer’s protocol. Samples were measured using a Varioskan LUX Multimode Microplate Reader (Thermo Fisher Scientific Inc., Waltham, MA, USA). Calcium deposits formed after CAH preparation were visualized by Alizarin Red staining. Representatives 10 and 50 mM CAHs were fixed in 4% PFA for 15 min, rehydrated, and incubated in 1% Alizarin Red for 10 min. Excess dye was removed, and samples were rinsed once with PBS. Optical images were acquired at 5× magnification using a Leica DM1 inverted microscope (Leica Microsystems GmbH, Wetzlar, Germany).

Scanning electron microscopy (SEM; Quanta 250, FEI) visualized CAH gel networks and calcium precipitation. Samples with or without NIH 3T3 cell seeding were fixed with 4% paraformaldehyde (PFA) in PBS on ice for 15 min. After fixation, samples were gently washed twice with PBS and sequentially dehydrated through a graded ethanol series (10, 30, 50, 70, 90, and 100%; 2 h per step). The ethanol-dehydrated CAHs were then dried using a critical point dryer (Leica EM CPD300, Leica Microsystems, Vienna, Austria) and mounted onto aluminum stubs using conductive tape. Subsequently, samples were sputter-coated with a platinum–palladium alloy for 30 s by using a sputter coater (Agar Scientific, Stansted, Essex, UK). Elemental analysis, via energy-dispersive spectroscopy (EDS), was performed using a FEI Quanta 3D 200i FIB-SEM fitted with an Oxford Instruments Xmas 50 mm^2^ SSD detector. The dried CAHs were mounted on aluminum stubs with colloidal graphite adhesive and then coated with a 10 nm Au/Pd layer, which provides sufficient surface grounding without fully suppressing the EDS signal from the specimen. EDS spectra were acquired at an acceleration voltage of 10 keV to efficiently excite the Ca Kα X-ray diffraction line (3.69 keV). Elemental silicon served as both intensity and energy calibration. A 75 μm × 75 μm scan area was used for all calibration and sample measurements.

X-ray diffraction (XRD) patterns of the CAHs were recorded using an Empyrean X-ray diffractometer (Malvern PANalytical, Almero, Netherlands) equipped with a Cu Kα radiation source (λ = 1.5406 Å) at an operating voltage of 45 kV and a current of 40 mA. Data acquisition employed a Bragg–Brentano reflection geometry with a 2θ range of 10–50° and a step size of 0.02°. FTIR spectra of CAHs were collected on a Thermo Scientific Nicolet iS50 FT-IR spectrometer equipped with an ATR accessory in the range of 4000–400 cm^–1^ at room temperature.

### 2.8 Statistical Analysis

Statistical analyses were conducted using SPSS software (ver. 17.0, SPSS Inc., Chicago, IL, USA). Data are presented as the mean ± standard deviation (SD) unless otherwise stated. Differences among multiple groups were evaluated by one-way analysis of variance (ANOVA), followed by Tukey’s post hoc test for pairwise comparisons. The significance level of *p* < 0.05 was considered statistically significant. The number of replicates and the significant differences between groups are indicated in the figures by asterisks as specified in the corresponding captions.

## 3 Results and Discussion

### 3.1 Fabrication and Characterization of CAHs

To investigate the cell–matrix interactions, we first established a series of hybrid hydrogels (CAHs) composed of collagen and alginate (Figure 1). Collagen was selected for its inherent cell-adhesive ligands, which are essential for supporting cellular adhesion and proliferation.^15^ However, pure collagen hydrogels possess inherently weak mechanical properties, and their stiffness can only be modulated within a narrow range by altering protein concentration.^21^ To overcome this limitation, alginate, which is a biocompatible and biologically inert polymer, was incorporated. Alginate can be readily cross-linked through ionic interactions via cytocompatible divalent ions (in this case, Ca^2+^) and has therefore become an established component in biomaterials research.^16^ By varying Ca^2+^ concentration from 2 to 50 mM during the ionic cross-linking, CAHs with broadly tunable elastic moduli have been developed. We tested all the hydrogels on a rotational rheometer, as well as a texture analyzer. As shown in Figure 2a, CAHs prepared with varying calcium concentrations exhibited storage modulus (*G*′) ranging from 200 to 6000 Pa, which falls within the mechanical strength range of most human soft tissues.^22^ The *G*′ of the 50 mM sample was about 30 times greater than that of the 2 mM sample. The compression tests on the texture analyzer also showed that the mechanical stiffness of the CAHs increased markedly with higher Ca^2+^ concentrations (Figure 2b). All samples exhibited typical hydrogel stress–strain behavior under compression. At 30% strain, CAHs treated with 50 mM calcium displayed the highest compressive stress (∼7 N), whereas hydrogel treated with 2 mM calcium showed less than 1 N. The increased stiffness can be attributed to the higher availability of Ca^2+^ ions, which coordinate with the negatively charged carboxyl groups of alginate chains. This promotes the formation of “egg-box”-like junctions with guluronate blocks in the alginate backbone, thereby enhancing cross-linking density and increasing gel rigidity.^23^

**Figure 2:**
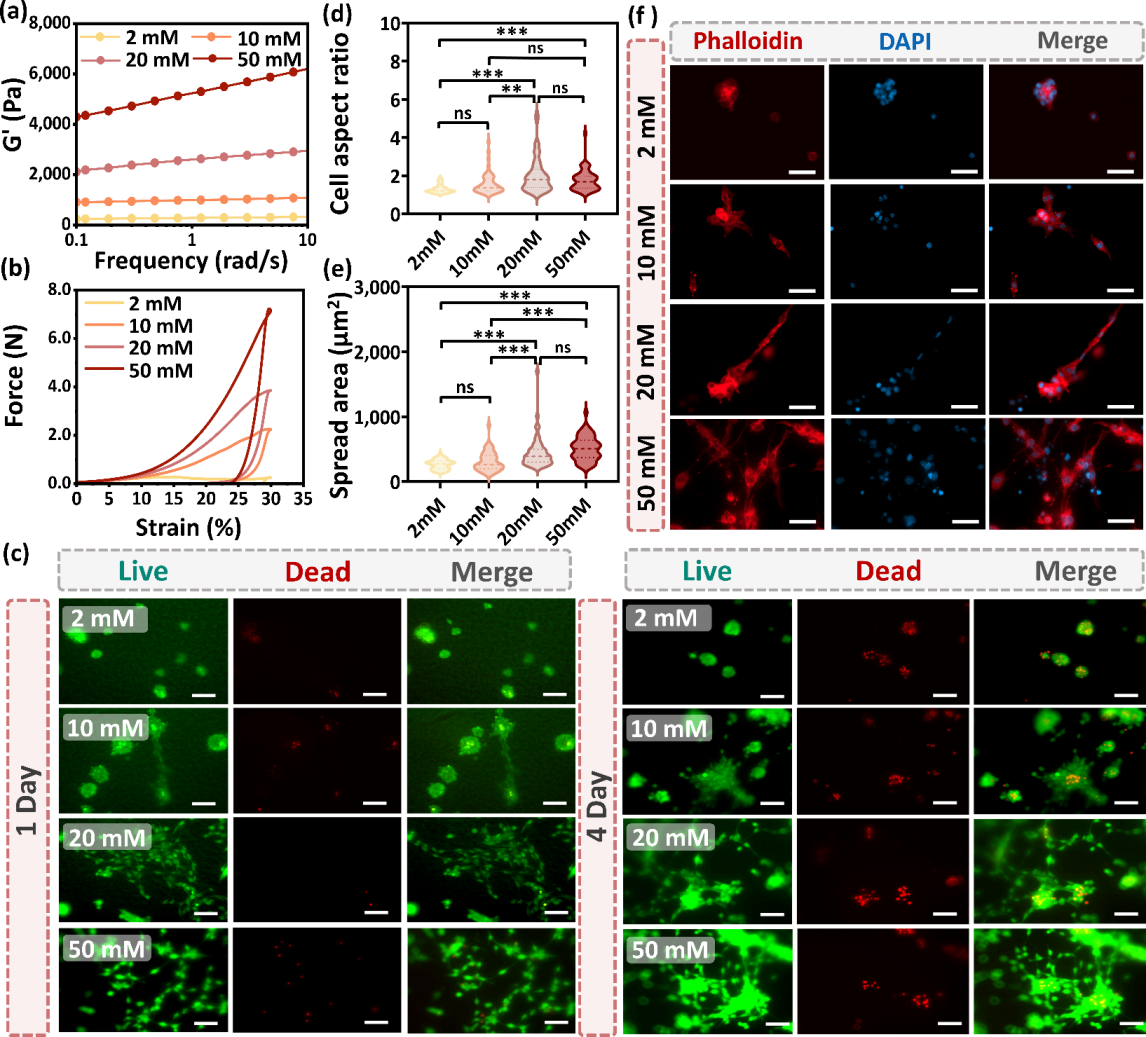
Physicochemical and biological characterization of CAHs prepared with different Ca^2+^ concentrations (2, 10, 20, and 50 mM). (a) Frequency sweep rheological analysis and (b) force-strain curves demonstrating tunable mechanical properties with increasing Ca^2+^ concentration. (c) Representative fluorescence images of live (calcein-AM, green) and dead (ethidium homodimer-1, red) NIH 3T3 cells cultured on CAHs at days 1 and 4. Scale bar: 100 μm. (d) Cell aspect ratio and (e) Quantification of cell spreading area of NIH 3T3 cells cultured on CAHs after 4 days. (f) Representative confocal images of cells cultured on CAHs of different stiffness for 4 days, stained for F-actin (Phalloidin-iFluor 594, red) and nuclei (DAPI, blue). Scale bar = 50 μm. Statistical significance: *p < 0.05, **p < 0.01, ***p < 0.001, ns = not significant.

Then we tested the cytocompatibility of the CAHs on a mouse embryonic fibroblast cell line (NIH 3T3). As illustrated in S-Figure 3, NIH 3T3 cells cultured on top of CAHs with varying stiffness maintained a live ratio above 90% after both 1 and 4 days, confirming the excellent cytocompatibility of CAHs. Fluorescence microscopy images of live/dead staining (Figure 2c) further support this observation, with strong green fluorescence indicating a predominance of live cells across all conditions. Furthermore, the cell morphology varied markedly with hydrogel stiffness. To visualize cell morphology, NIH 3T3 cells were stained with DAPI and phalloidin to label nuclei and actin filaments, respectively, and imaged by confocal microscopy (Figure 2f). On the softer 2 mM CAHs (*G*′ ≈ 200 Pa), cells exhibited limited spreading and remained mostly spherical or aggregated into clusters, indicating restricted ductility. In contrast, on the stiffer 20 and 50 mM CAHs (*G*′ ≈ 2000 and 6000 Pa, respectively), cells adopted an elongated morphology with greater spreading areas, resembling those observed on conventional tissue culture plates. Consistently, the average cell spreading area increased from ∼250 μm^2^ with an aspect ratio of ∼1 on soft CAHs to ∼500–1000 μm^2^ with aspect ratios exceeding 2 as matrix stiffness increased (Figure 2d,e). This stiffness-dependent morphological response is consistent with previous reports showing that substrate mechanics strongly influence cytoskeletal organization and cell adhesion.^24,25^ The spherical morphology observed in softer CAHs may limit focal adhesion formation, thereby hindering cell extension, while stiffer matrices provide more favorable conditions for actin remodeling and spreading. Given the results shown above, it is concluded that CAHs with tunable stiffness are suitable for cell–matrix interaction studies.

### 3.2 Mechanical Stability of CAHs

To study cell–matrix interactions on a biorelevant time scale (from days to weeks), we investigated whether CAHs could maintain their mechanical properties during the studied period. Specifically, we soaked the prepared CAHs in biorelevant buffer PBS and DMEM culture medium supplemented with 10% FBS (FM). Their mechanical properties were characterized after 1 and 4 days. As displayed in Figure 3, compared with freshly prepared samples, CAHs in both PBS and FM exhibited significant reductions in stiffness, with the greatest decrease observed in PBS. We assumed that the loss of stiffness could be attributed to ion exchange during culture, whereby sodium, potassium, magnesium, and phosphate ions progressively displace calcium cross-links within the alginate-based network, leading to scaffold degradation.^26^ Earlier studies revealed that the Ca^2+^ in the medium can mitigate the calcium deprivation effect and stabilize alginate hydrogels, but our results indicate that this effect is no longer effective when the initial calcium treatment concentration exceeds 10 mM.^27,28^

**Figure 3:**
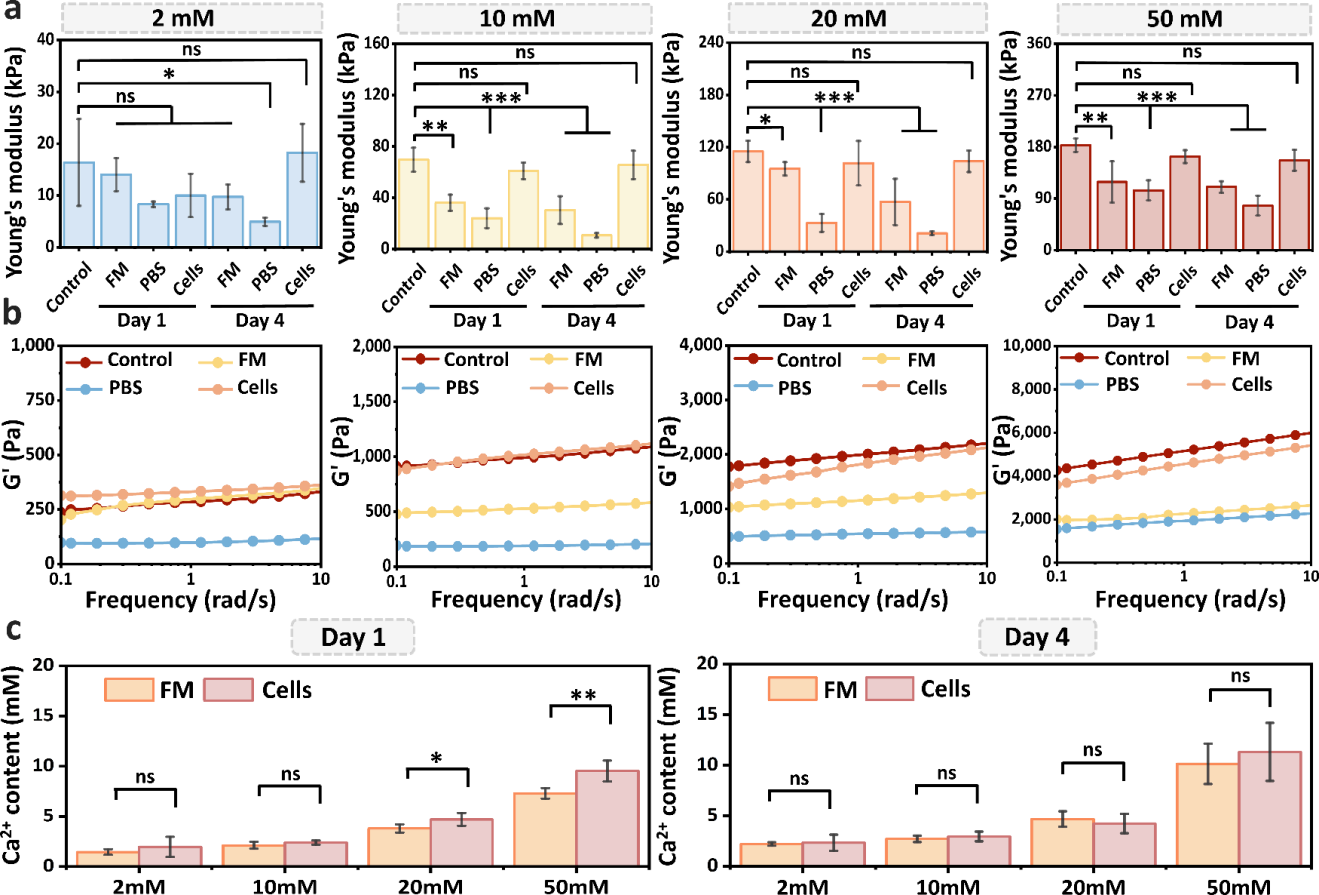
Mechanical properties and calcium release of CAHs under different culture conditions. (a) Young’s modulus of CAHs cross-linked with 2, 10, 20, or 50 mM Ca^2+^ after 1 or 4 days of incubation in fibroblast medium (FM), phosphate-buffered saline (PBS), or FM containing NIH 3T3 fibroblasts (Cells). (b) Frequency sweep rheological profiles of CAHs cultured for 4 days under the same conditions as in (a). (c) Free Ca^2+^ concentrations measured in the supernatant after 1 and 4 days of culture in FM or FM with NIH 3T3 fibroblasts (Cells). Data are presented as mean ± SD. Statistical significance: *p < 0.05, **p < 0.01, ***p < 0.001, ns = not significant.

Surprisingly, we observed that CAHs seeded with NIH 3T3 cells showed almost identical stiffness compared with freshly prepared CAHs. As shown in the frequency sweep data (Figure 3b and Figure S4), whereas gels cultured in PBS or FM alone exhibited obvious decreases in stiffness, cell-laden gels maintained a *G*′ comparable to that of the freshly prepared gels after both 1 and 4 days. Given the unexpected results, we did an extensive literature search on alginate hybrid hydrogels and how their stiffness varied during cell culture. So far, most previous studies have primarily emphasized Ca^2+^ loss from alginate networks during culture and the impact of gel stiffness on cell behavior, but the potential effects of cell seeding on gel mechanical stability have been less explored.^29,30^ The only existing studies were major studies conducted in 3D culture systems, and the results were inconsistent. For example, Hunt et al. reported that the *G*′ of cell-laden alginate hydrogels decreased more rapidly than that of decellularized controls.^26^ They attributed this to cellular utilization of residual Ca^2+^, which reduces the ions available for cross-linking. Another study on alginate microgels found that, apart from modulus reduction due to swelling, no significant decrease in stiffness was observed after up to 8 days of culture.^31^ In contrast, Williams et al. found that increasing chondrocyte density within alginate constructs enhanced extracellular matrix mechanical strength, which may be related to matrix accumulation.^32^

Based on the literature findings discussed above, we first hypothesized that cellular activity might reduce Ca^2+^ loss from the gel, thereby inhibiting the mechanical degradation of the network. To examine this hypothesis, the Ca^2+^ release from CAHs in FM with or without cell seeding was quantified to evaluate the potential contribution of cells in limiting calcium loss. As shown in Figure 3c, free Ca^2+^ concentrations released from the CAHs to the supernatant increased over time across all groups, indicating time-dependent calcium leaching from all hydrogels. The same results were also observed under the PBS conditions (Figure S5). Notably, under FM conditions, cell-laden and decellularized CAHs exhibited comparable calcium release. Furthermore, CAHs cross-linked with higher Ca^2+^ concentrations released larger amounts of Ca^2+^ upon immersion in FM, indicating that calcium release is also governed by concentration-gradient-driven diffusion, whereby larger differences between internal and external calcium levels accelerate leaching. Therefore, Ca^2+^ release from CAHs is primarily governed by concentration-gradient-driven diffusion, in which Ca^2+^ migrates outward along the gradient and is not significantly influenced by cellular activity. This observation rules out the hypothesis that cells effectively inhibit the Ca^2+^ loss from CAHs. Accordingly, we further propose that the cell-induced enhancement of mechanical stability might be instead associated with modifications to the network structure of the CAHs.

### 3.3 Micro- and Macroscopic Structure of CAHs

To further elucidate the potential relationship between cellular effects and the CAH network structure, we first imaged hydrogels cross-linked with different Ca^2+^ concentrations prior to cell seeding. Micro- and macroscopic structures of CAHs were imaged by SEM at both 4000× and 500× magnifications, respectively, following supercritical drying, a procedure that better preserves the native hydrogel microstructure. As shown in Figure 4a, at high magnification, all CAH sections displayed a relatively homogeneous gel network with no visible differences in microstructural morphology among samples. This indicates that although the extent of ionic cross-linking increases with Ca^2+^ content, such variations are not readily distinguishable from the pore architecture preserved in SEM. In contrast, low-magnification SEM images revealed distinct surface-associated deposits (arrows in Figure 4b), which became more prominent at higher calcium concentrations. This observation may suggest that micrometer-scale insoluble deposits are generated during CAH preparation and that these Ca^2+^-DMEM-derived precipitates are predominantly retained at the surface layer, without altering the morphology of the underlying internal network. At 10, 20, and 50 mM, white precipitates were clearly visible, whereas the 2 mM gels remained largely free of deposits. This indicates that the quantity of precipitates is positively related to the initial cross-linking concentration. However, we cannot exclude the possibility that some calcium aggregates formed during SEM sample drying. To verify that the observed deposits pre-existed prior to SEM preparation, we stained representative 10 and 50 mM CAH systems with Alizarin Red. As illustrated in Figure S6, both appearance imaging and optical microscopy clearly revealed intense Alizarin Red-positive deposits at the surface layer of the 10 and 50 mM CAHs, whereas CAHs with Ca-induced cross-linking only exhibited a light red background signal. After ethanol washing, the amount of surface deposits on SEM samples was markedly reduced compared with freshly prepared CAHs, indicating that these calcium deposits are not covalently bonded and can be partially lost due to liquid flow.

**Figure 4:**
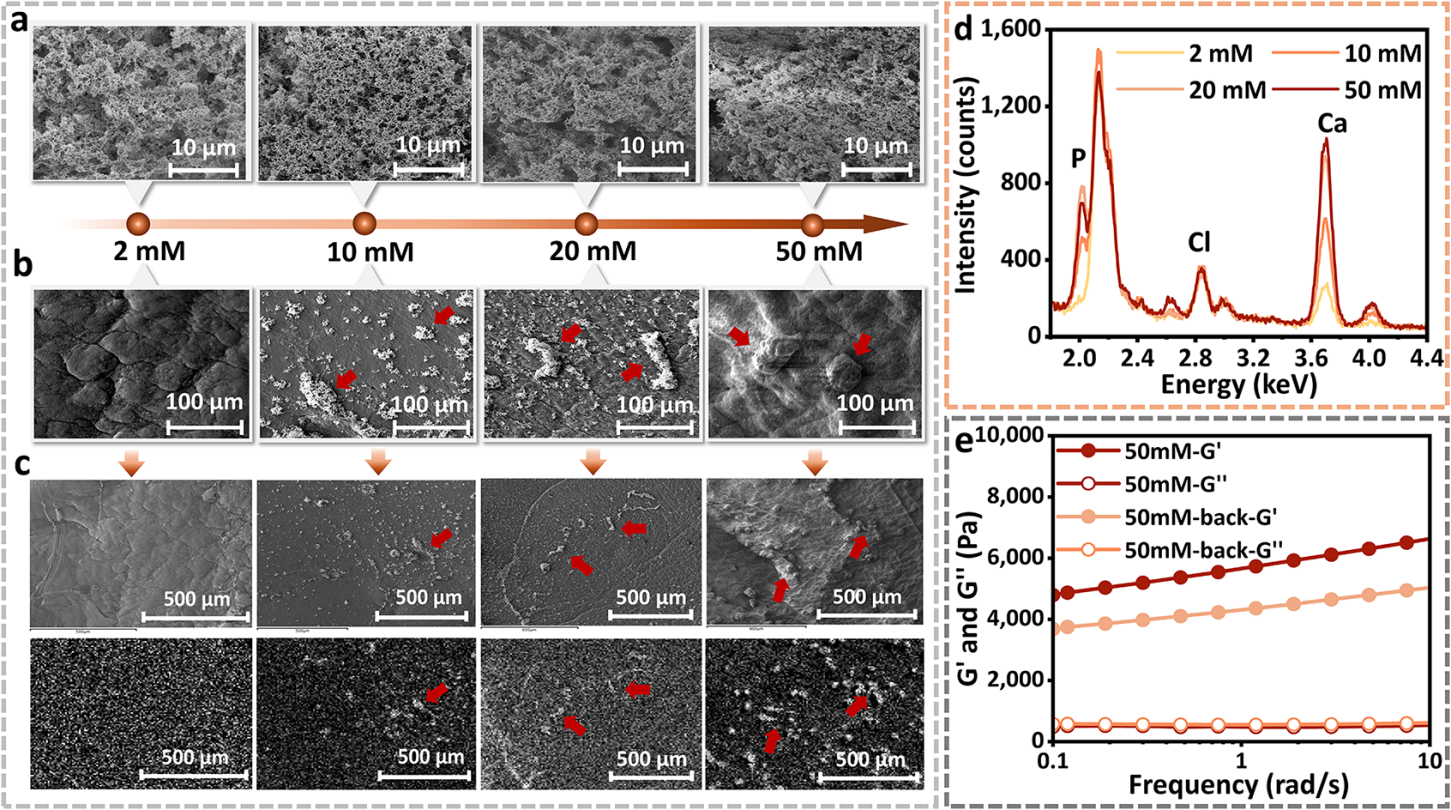
Microscopic characterization and calcium distribution of CAHs cross-linked with varying calcium concentrations. (a) Representative SEM nanoscale gel network structures of supercritical dried CAHs cross-linked with 2, 10, 20, or 50 mM Ca^2+^ at 4000× magnification. (b) Representative SEM micrographs (500×) of CAH surfaces prepared with 2, 10, 20, and 50 mM Ca^2+^. Red arrows denote the progressive appearance of surface-associated mineral deposits with an increasing Ca^2+^ concentration. (c) SEM images of CAHs cultured in FM showing mineral deposits on hydrogel surfaces (top) and corresponding EDS calcium elemental maps (bottom). Red arrows indicate regions with enriched Ca signals. (d) EDS spectra of surface deposits formed on CAHs cross-linked with 2, 10, 20, and 50 mM Ca^2+^. (e) Frequency sweep rheological analysis of untreated 50 mM CAHs comparing the mineral-rich front surface and mineral-free back surface.

To further characterize the chemical composition of these deposits, we performed EDS mapping was performed. As shown in the elemental maps (Figure 4c), regions of high calcium signal overlapped precisely with the observed surface precipitates, while the surrounding gel matrix exhibited much lower intensities. This strong spatial correlation indicates the high calcium concentration of the deposits. Notably, the calcium precipitates formed under the 10 and 20 mM treatments appeared as amorphous aggregates, whereas the 50 mM treatment yielded deposits with irregular crystalline morphology. This may be related to the ion concentration of the system and the composition of the deposits. Generally, under physiological conditions, calcium solutions with relatively low supersaturation do not readily crystallize into carbonate-doped hydroxyapatite upon mixing with phosphate and carbonate ions.^33,34^ Instead, they initially generate an amorphous precursor, which can persist as disordered intermediates for extended periods.^35^ Such long-standing existence of amorphous nanogranular in calcium phosphate or calcium carbonate mineralized collagen-based and alginate-based hydrogel systems is well established.^36–39^ However, at the higher Ca^2+^ concentrations of 50 mM sample, the existing Ca^2+^ far exceeds that at the mineral deposition site, preventing sustained stabilization of amorphous precursor and accelerating its transformation into more thermodynamically stable crystalline phases.^40^

It is worth noting that, in addition to the contribution of Ca^2+^, collagen in CAHs is also regarded as a passive scaffold and template for mineral formation. CaP mineralization has been reported to occur within the nanoporous architecture generated by the specific fibrillar arrangement of collagen molecules.^41^ As further evidence, we obtained EDS spectra from CAH surface regions that intentionally avoided large surface aggregates (Figure 4d). As expected, the Ca and P signals within the surface layer increased progressively from 2 to 50 mM Ca^2+^ cross-linking concentrations, indicating greater incorporation of Ca- and P-containing species into the surface layer at higher Ca^2+^ levels. Notably, the Cl peak (∼2.6 keV) on the 50 mM CAH surface was substantially higher than that on the 20 mM CAH surface. This observation suggests that when the Ca^2+^ concentration exceeds a threshold (>20 mM), the available phosphate becomes depleted, leading to the incorporation of Cl^–^ into the precipitated phase. Accordingly, the deposits formed on the 50 mM CAHs are likely mixed Ca–P–Cl mineral species. Overall, the abundance of calcium-based precipitates can be attributed to two intrinsic properties of CAHs: (i) the high content of Ca^2+^ and anions from the Ca^2+^-DMEM cross-linking solution establishes supersaturation conditions that drive the transition of ions from solution to solid mineral phases; (ii) the collagen present in the matrix acts as a template to provide nucleation sites for deposition and guiding the nucleation and directional growth of calcium deposits.^42,43^

The complex deposition observed in the CAH system demonstrates that Ca^2+^ ions within the system not only induce cross-linking of the alginate hydrogel network but also drive calcium deposition at the gel–medium interface. Similar spontaneous CaP deposition on calcium alginate hydrogels incubated in phosphate-containing media has been reported previously, supporting our observations.^44,45^ Inevitably, the presence of calcium deposits introduces macroscopic calcium heterogeneity into the system, potentially altering the system’s mechanical properties. To evaluate the mechanical impact of surface CaP deposition, rheological tests were conducted on representative 50 mM CAHs prior to FM immersion, and the mineral-rich front surface and mineral-free back surface were tested separately (Figure 4e). As expected, the mineral-free surface of 50 mM CAHs was significantly softer than the mineral-rich front surface, indicating that mineral deposition at the gel–medium interface provided localized mechanical reinforcement.

As a summary, CAHs with distinct mechanical properties but similar micronetwork structures display markedly different calcium mineral accumulation. These observations prompted us to revise our previous hypothesis and suggest that cell-induced mechanical stabilization is linked to calcium deposition.

### 3.4 Cell–Mineral Interactions

To further understand the redistribution of calcium deposition in the CAH system during cell culture, SEM was employed to examine the surface of the hydrogel after cell seeding. As shown in Figure 5a, after the Ca-DMEM cross-linking solution was replaced with FM containing NIH 3T3 fibroblasts, new calcium-based deposition emerged on the hydrogel surface. Combined with the results of free calcium content detected in the supernatant, it can be inferred that only a fraction of Ca^2+^ released from the hydrogel remains dissolved in FM, while the majority is consumed through the formation of insoluble calcium deposits. In softer CAHs, where the limited Ca^2+^ availability restricted deposition, only sparse deposits were detected with a limited number of spreading fibroblasts. With increasing gel stiffness and greater calcium availability, pericellular mineral-like deposits became more abundant, accompanied by pronounced cytoskeletal extensions. At the highest cross-linking concentration (50 mM), fibroblast surfaces were densely associated with mineral aggregates, which formed continuous clusters and largely obscured the underlying matrix. This preferential juxta-cellular enrichment of calcium deposits implies the localized coupling between fibroblast activity and mineral deposition.

**Figure 5:**
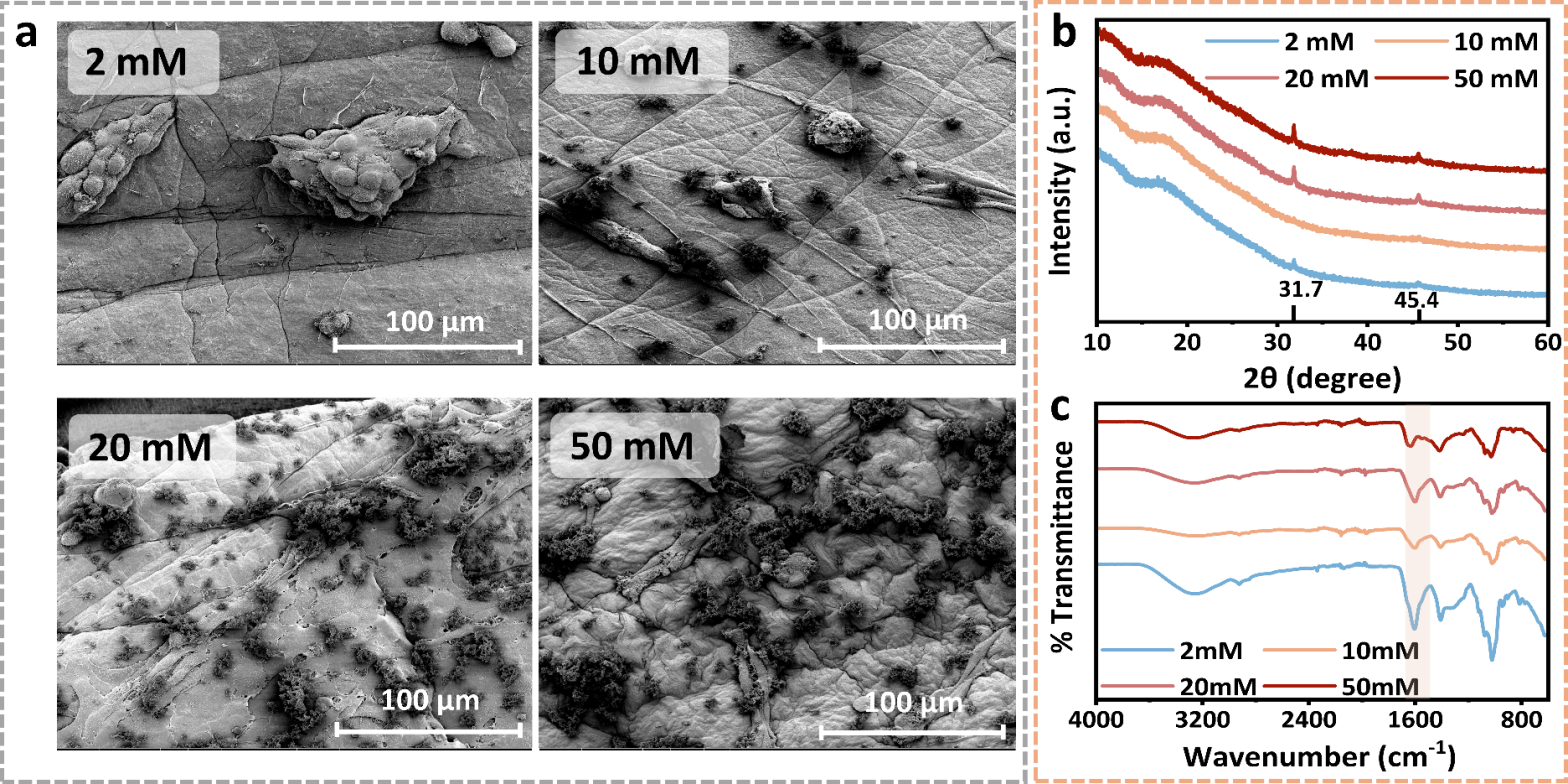
Physicochemical characterization of Ca^2+^ deposits formed during cell culture. (a) SEM micrographs of surface Ca^2+^ deposits on CAHs cross-linked with 2, 10, 20, and 50 mM Ca^2+^ after 4 days of culture with NIH 3T3 cells. Scale bar = 100 μm. (b) XRD and (c) FTIR spectra of CAHs after 4 days of culture within FM. Highlighted regions correspond to the COO^–^ asymmetric stretching band (∼1400–1600 cm^–1^) in the FTIR spectrum.

To investigate the nature of calcium deposits formed during cell culture, we attempted to perform XRD and FTIR tests on various CAHs. As shown in Figure 5b, the XRD patterns were dominated by broad diffraction features from collagen and alginate, along with distinct crystalline peaks corresponding to residual NaCl from FM, making it difficult to resolve any potential mineral phase. Notably, no sharp diffraction peaks attributable to crystalline calcium phosphate phases (e.g., hydroxyapatite or octacalcium phosphate) were observed, implying that CaP deposition in the CAHs system is highly likely to exist in amorphous form. FTIR spectra of CAHs showed a gradual decrease in the COO^–^ asymmetric stretching band (∼1400–1600 cm^–1^) with increasing Ca^2+^ concentration, indicating enhanced Ca^2+^-alginate coordination consistent with “egg-box” cross-linking (Figure 5c). However, no well-resolved PO_4_^3–^ stretching band (typically ∼1030–1100 cm^–1^) could be distinguished from the polymer background. This is likely due to strong spectral overlap between the phosphate ν_3_ vibration and the C–O–C stretching bands of alginate in the same region, as well as the inherently broad PO_4_ absorption associated with amorphous calcium phosphate (ACP). It has been revealed that spontaneous precipitation of ACP can occur in serum-containing DMEM due to supersaturation effects upon Ca^2+^ increase.^46,47^ Serum-derived organic polyanions and proteins, particularly potent mineralization inhibitors such as Fetuin-A and phosphorylated glycoproteins, could strongly bind calcium and stabilize amorphous precursor phases.^48,49^ These molecules act by forming calciprotein particles (CPPs) and by adsorbing onto nascent nuclei, thereby lowering surface free energy, extending the induction period, and suppressing rapid crystallization.^50^

The existence of calcium deposition demonstrates that Ca^2+^ released from CAHs was sufficient to achieve supersaturation. Together with nucleation sites provided by the collagen-containing gel matrix, these mineralization promoters transformed CAHs from a conventional 2D culture substrate into a potentially mineralization-promoting hydrogel platform resembling established in vitro models.^51^ Within the diffusion-restricted hydrogel environment, Ca^2+^ released from the gel preferentially establishes supersaturation at the gel–medium interface, where it interacts with inorganic and organic anions in the culture medium, resulting in surface-confined discontinuous ACP formation. During this process, the cell membrane composed of negatively charged units (e.g., phospholipids, glycoproteins, and glycosaminoglycans) of NIH 3T3 cells also sequesters Ca^2+^, generating localized calcium-enriched regions that act as potential deposition sites. Furthermore, ECM components secreted by fibroblasts, such as type I collagen, furnish nucleation templates that facilitate the deposition of subsequent amorphous precursor phases.^41,52,53^ Meanwhile, the variety of Ca^2+^ transporters and channels of fibroblasts that continuously absorb and release calcium ions may potentially form a localized gradient around the cell membrane. Therefore, such cell-induced high Ca^2+^ concentration microenvironment may act as “anchors” to drive calcium deposition preferentially near cells.^42,54,55^

### 3.5 Microscale Matrix Mechanics by AFM

To further examine the relationship between cell-associated calcium deposition and matrix mechanical strength during culture, AFM nanoindentation was performed to characterize the micrometer-scale Young’s modulus of representative 50 mM CAHs. Representative force–indentation curves of cell-free and cell-seeded gels are shown in Figure S7. As expected, CAHs without cell seeding (softer hydrogels) allowed deeper probe indentation under the same applied force. To assess the microscale mechanical heterogeneity of CAHs, four regions were randomly selected on the surfaces of gels with and without cells (Figure 6a). Each region was probed by AFM nanoindentation force mapping, comprising 256 indentation points within an area of 100 μm^2^. The modulus maps revealed that cell-free CAHs exhibited darker distributions, indicating lower stiffness values, which is consistent with bulk rheological measurements. By contrast, CAHs cultured with fibroblasts displayed overall brighter maps, reflecting increased stiffness, with distinct localized regions of markedly elevated modulus. To validate these observations, modulus distributions obtained from over 1000 indentation points were statistically compared between 50 mM CAHs with and without NIH 3T3 fibroblasts. As expected, cell-seeded CAHs exhibited markedly higher stiffness overall (Figure 6 b). After 4 days in FM, cell-free CAHs displayed relatively uniform stiffness, with Young’s modulus values ranging from 0.10 to 8.29 MPa. By contrast, cell-laden CAHs showed a much broader distribution (1.12–78.22 MPa), indicating the presence of significant micrometer-scale mechanical heterogeneity. This heterogeneity might be attributed to two main factors: (i) collagen rearrangement and densification driven by actomyosin-mediated cellular traction and (ii) calcium deposition within the system. Previous studies have reported that in the fibrillar type I collagen hydrogels, the traction forces exerted by migrating cells align and stretch collagen fibers, generating localized strain stiffening within a zone extending up to 100 μm in front of the leading edge.^56^ Likewise, Viji Babu et al. demonstrated under 2D conditions that fibroblast-ECM mechanics are bidirectionally coupled, with cells guided by the matrix while simultaneously remodeling and altering its local mechanical properties.^57^ In addition, the pericellular mineral aggregates that form around cells are likely to possess a stiffness higher than that of the underlying hydrogel matrix, further broadening the modulus distribution. Therefore, in this mineralization CAH system, the alginate network serves as a sustained source of calcium ions, the collagen matrix provides an organized template for nucleation and mineral growth, and fibroblasts act as active anchors that both remodel collagen fibrils and influence the distribution of calcium deposits. The combined contributions of ionic release, matrix templating, and cell-mediated remodeling establish a reinforced microenvironment that helps stabilize the mechanical properties of the hydrogel.

**Figure 6:**
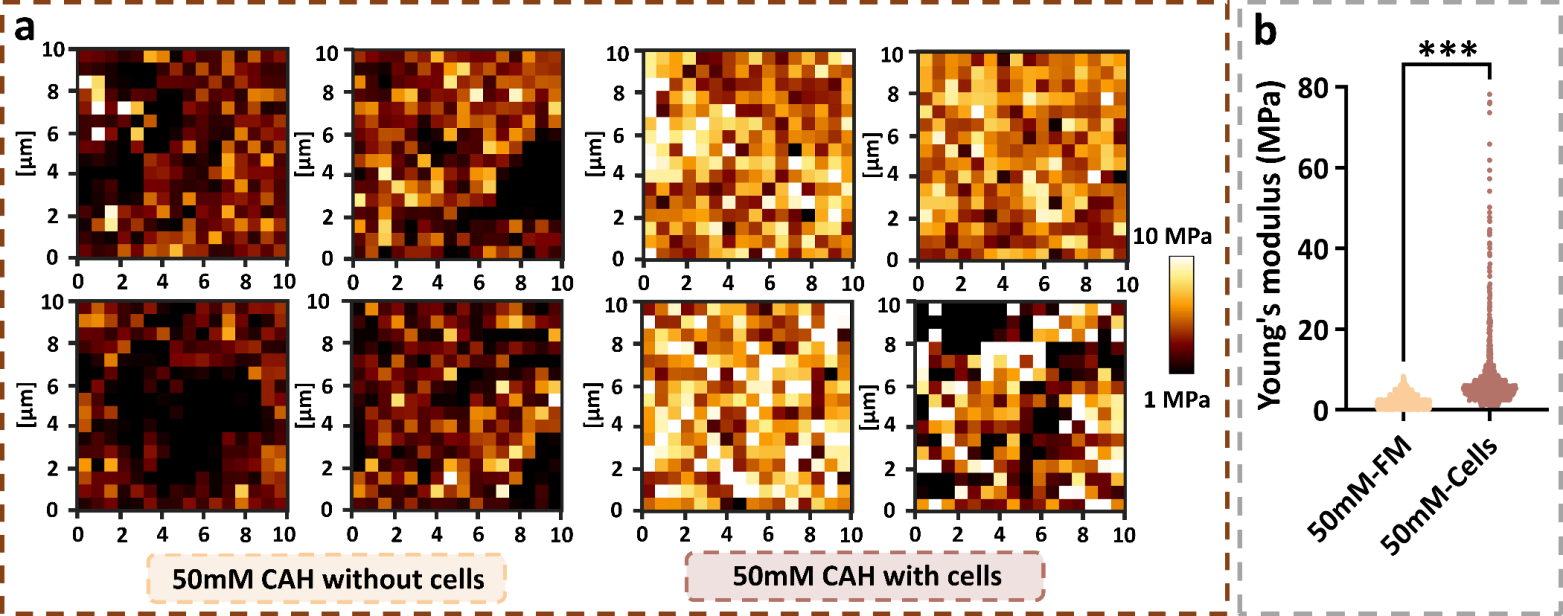
Micromechanical characterization of representative CAHs) with or without NIH 3T3 fibroblasts. (a) Force maps at four random surface locations of CAHs (50 mM Ca^2+^) cultured with or without cells. The color bar represents the Young’s modulus. (b) Statistical comparison of surface Young’s modulus distributions (*n* > 1000 indentation points) for 50 mM CAHs cultured in fibroblast medium (FM) with or without NIH 3T3 cells for 4 days (***p < 0.001).

### 3.6 Bulk Mechanics Governed by Cell–Calcium Interactions

Despite the AFM results revealing the relationship between cell-associated calcium deposition and matrix mechanical strength on the micrometer scale, it remains unclear whether such localized reinforcement translates into macroscopic mechanical effects. To address this question, we further investigated the role of cells, calcium deposition, and their interplay at the macroscopic scale using a rotational rheometer. First, we seeded 20 mM CAHs with varying densities of NIH 3T3 cells and cultured them in FM for 4 days before measuring their bulk *G*′ (Figure 7a). The results revealed that maintenance of mechanical strength was evident only when cell density exceeded a threshold, whereas gels containing no cells or only ∼10^2^ cells showed a marked decrease in *G*′. For NIH 3T3 fibroblasts, the typical projected area of an attached and spread single cell under 2D culture conditions is reported to be approximately 900–2500 μm^2^.^58^ Accordingly, when more than ∼10^4^ cells are seeded onto the CAH surface (≈3.8 cm^2^ surface area in a 12-well plate), the estimated surface coverage is >5–13% after 1 day of culture and increases to >40–75% after 4 days. These findings suggest an essential cell density or surface coverage is required for fibroblasts to function as effective “mechanical anchors” to maintain the macroscale stiffness of CAHs.

**Figure 7:**
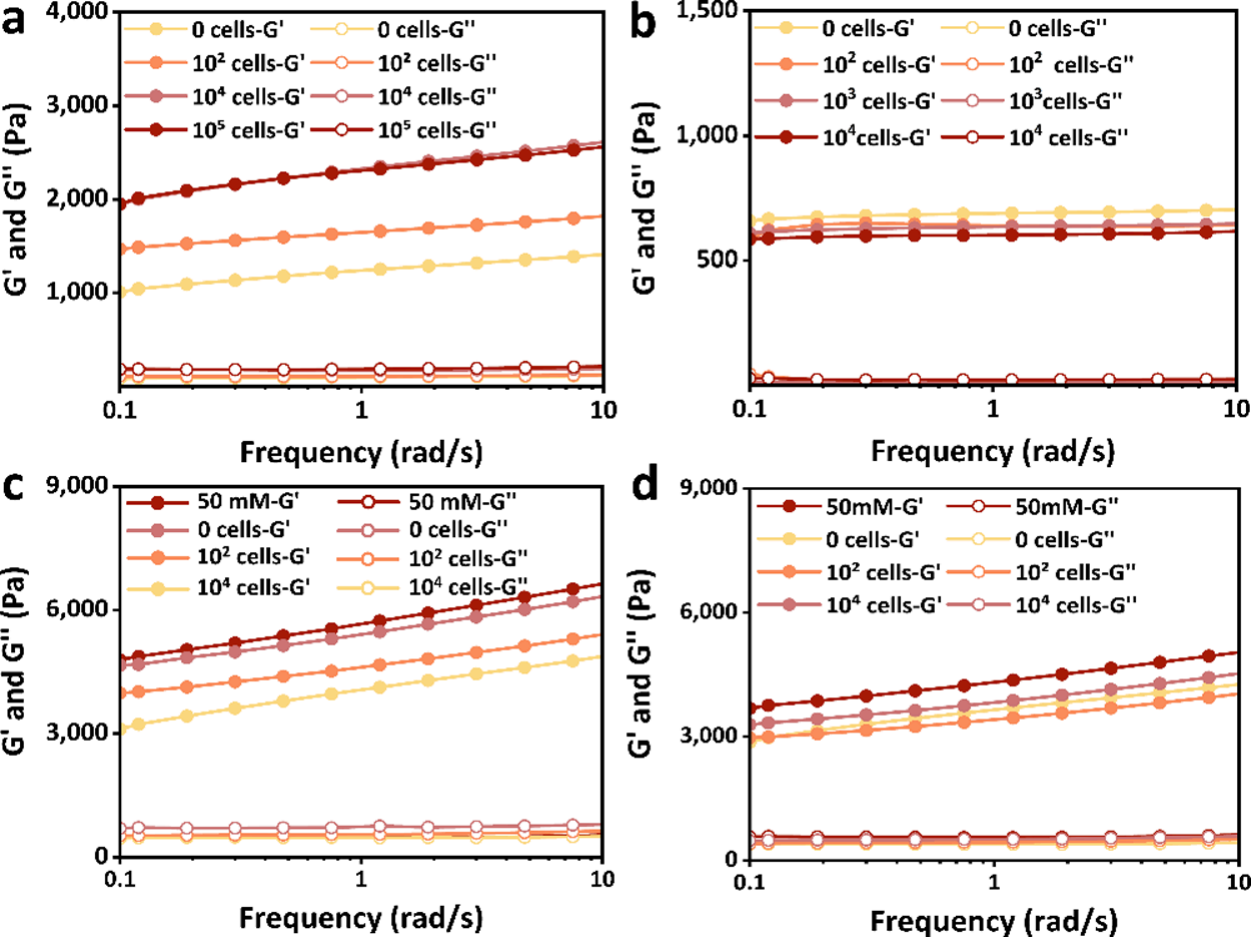
Rheological Characterization of CAHs seeded with NIH 3T3 fibroblasts at varying densities. (a) Frequency sweep rheological analysis of bulk CAHs cross-linked with 20 mM Ca^2+^ containing 0, 10^2^, 10^4^, or 10^5^ cells per hydrogel. (b) Frequency sweep rheological analysis of Tz-Norb covalently cross-linked CAHs cultured for 4 days in fibroblast medium (FM) with different cell densities (0, 10^2^, 10^3^, and 10^4^ cells). (c) Frequency sweep analysis of the mineral-rich front surface of 50 mM CAHs seeded with different cell densities (0, 10^2^, and 10^4^ cells). (d) Frequency sweep analysis of the mineral-free back surface of the same CAHs described in (c). Untreated 50 mM CAHs without cells were used as the controls.

After confirmation of the contribution of cells, control CAHs lacking calcium deposition were prepared to further verify whether mineral deposition is indispensable for maintaining mechanical stability. The control CAHs were fabricated through covalent cross-linking of Tz-modified alginate with Norb-modified alginate, in combination with collagen self-assembly, to yield a comparable hydrogel network. Successful functionalization of alginate was verified by ^1^H NMR and FTIR spectroscopy (Figure S1). The appearance of a new absorption band at ∼1732 cm^–1^, corresponding to the C=O stretching vibration of ester/amide linkages, confirms successful grafting.^59^ Based on ^1^H NMR analysis, the degrees of substitution of Tz-alginate and Norb-alginate were determined to be 10% and ∼6.1%, respectively. NIH 3T3 cells were then seeded at varying densities to evaluate whether cell-induced effects alone could confer macroscopic mechanical changes. As displayed in Figure 7b, the *G*′ values of Tz-Norb CAHs remained comparable across all cell densities, with no significant differences observed between groups. These findings confirm that the micrometer-scale stiffness enhancement induced by fibroblast-mediated compaction of the surrounding matrix does not translate into measurable macroscopic mechanical reinforcement of the hydrogel if the calcium deposition is absent.

As noted above, calcium deposition within the CAH system mainly accumulates at the hydrogel–medium interface (front surface) and does not penetrate the hydrogel bulk. Thus, to further elucidate the mechanical contribution of this asymmetric mineralization, we compared the *G*′ of the mineral-rich front surface with that of the mineral-free back surface in representative 50 mM CAHs. As illustrated in Figure 7 c and d, the *G*′ values of cell-seeded CAHs were consistent with previous results. Untreated 50 mM CAHs exhibited the highest *G*′ on the calcium-rich surface, comparable to that of CAHs seeded with 10^4^ cells. Lower values were observed for CAHs seeded with 10^2^ cells, while cell-free CAHs showed the lowest *G*′. Comparison of the front and back surfaces revealed that the mineral-free surface of CAHs was markedly softer than the mineral-rich surface, highlighting the mechanical reinforcing effect of surface-associated calcium precipitates. It is worth noting that after 4 days of cell culture, the back surface of CAHs without calcium precipitate showed similar *G*′ values, regardless of whether cells were present, and all of them were lower than the *G*′ values of the back of freshly prepared CAHs. This suggests that, irrespective of calcium precipitation, both cell-seeded and cell-free gels experienced comparable mechanical degradation, implying a similar extent of calcium loss and supporting our previous findings. Therefore, it could be demonstrated that the mechanical stability of CAHs only acts on the mineral-rich surface. These findings highlight that both calcium precipitation and sufficient cell density are indispensable for sustaining the mechanical stability of CAHs. This result also supports our second hypothesis that the transformation of “disappeared” calcium ions into surface mineral deposits, together with the presence of cells, synergistically contributes to maintaining the mechanical stability of CAHs.

## 4 Conclusions

In this study, we systematically investigated the mechanical stability of CAHs under different culture conditions (PBS, FM, or FM with NIH 3T3 cells). Our results demonstrate that CAHs are broadly cytocompatible and can be tuned across a wide stiffness range with Ca^2+^ treatment. However, in both PBS and FM environments, their mechanical stiffness decreases markedly after culture due to ion exchange and osmotic equilibration with the surrounding medium. Notably, the presence of cells significantly suppressed this instability of the gel, indicating positive interactions between the cells and matrix.

In FM, continuous calcium release combined with the collagen component of CAHs promotes the formation and accumulation of stabilized amorphous calcium precursors at the gel–medium interface, leading to extensive surface CaP deposition. In this case, CAHs no longer behave as simple 2D substrates but instead function as a potentially mineralization hydrogel platform. Furthermore, calcium deposits that emerge during culture have been shown to exhibit spatial association with cells, and cell-mediated reinforcement of gel stiffness is achieved only through synergy with calcium deposits. Importantly, this effect is confined to the mineral-rich surface layer and becomes significant only at sufficiently high cell densities, where fibroblasts act as both nucleation sites and mechanical anchors.

Collectively, these findings establish CAHs as dual-function systems, serving both as mechanically tunable hydrogels and as a potential mineralization platform. Importantly, our results demonstrate that in a 2D environment, fibroblasts not only respond to substrate mechanics but also influence the properties of the matrix itself. By clarifying the interplay among calcium release, mineral deposition, and cell–matrix interaction, this study advances the understanding of the mechanical instability inherent to alginate-based hydrogels and provides guidance for their rational design in tissue engineering applications.

## Supplementary Material



## Notes

^1^H NMR spectra and FTIR spectra of functionalized alginate, Additional rheological characterization, Cell viability, Calcium release analysis, Alizarin Red staining of surface deposition, and AFM force–displacement measurements of hybrid hydrogels (PDF)
