# Supplementary material for: Cell-Mediated Mechanical Stability Enhancement of Biomimetic Collagen-Alginate Hydrogels: A Mechanistic Study on the Two-Dimensional Extracellular Matrix–Cell Interaction
Source: Chem Mater. 2026 Apr 6;38(8):4115–27. doi: 10.1021/acs.chemmater.5c03410 (PMC13130400; doi:10.1021/acs.chemmater.5c03410)
Supplement: Supplementary file 1 [file cm5c03410_si_001.pdf]

Supporting Information for

Cell-mediated mechanical stability enhancement of biomimetic collagen-alginate hydrogels: a mechanistic study on the 2D extracellular matrix-cell interaction

Shuhan Feng[1,2], Sami Hietala[3], Juan José Valle-Delgado [4], Marko Vehkamäki[3], Alexandra Correia[1], Shiqi Wang[1,2*]

Affiliations

1 Drug Research Program, Divisions of Faculty of Pharmacy, University of Helsinki, Helsinki, FI-00014, Finland.

2 Institute of Biotechnology, Helsinki Institute of Life Science (HiLIFE), University of Helsinki, FI-00014, Helsinki, Finland.

3 Department of Chemistry, Faculty of Science, University of Helsinki, FI-00014, Helsinki, Finland.

4 Department of Bioproducts and Biosystems, School of Chemical Engineering, Aalto University, FI-00076 Aalto, Finland.

Email: shiqi.wang@helsinki.fi

This PDF file includes:

Supplementary Figure 1. Chemical characterization of functionalized alginates.

Supplementary Figure 2. Strain-sweep rheological analysis of collagen-alginate hybrid hydrogels (CAHs).

Supplementary Figure 3. Quantification of live ratios of NIH 3T3 fibroblasts cultured on CAHs after 1 and 4 days.

Supplementary Figure 4. Frequency sweep rheological analysis of CAHs after 1 day under different culture environments

Supplementary Figure 5. Relative calcium ion release from CAHs crosslinked with different Ca^{2+} concentrations after incubation in fibroblast medium and PBS.

Supplementary Figure 6. Alizarin Red staining of surface mineralization in 10- and 50-mM CAHs.

Supplementary Figure 7. Representative AFM force-displacement curves of CAHs crosslinked with 50 mM Ca^{2+}, with or without NIH 3T3 fibroblasts.

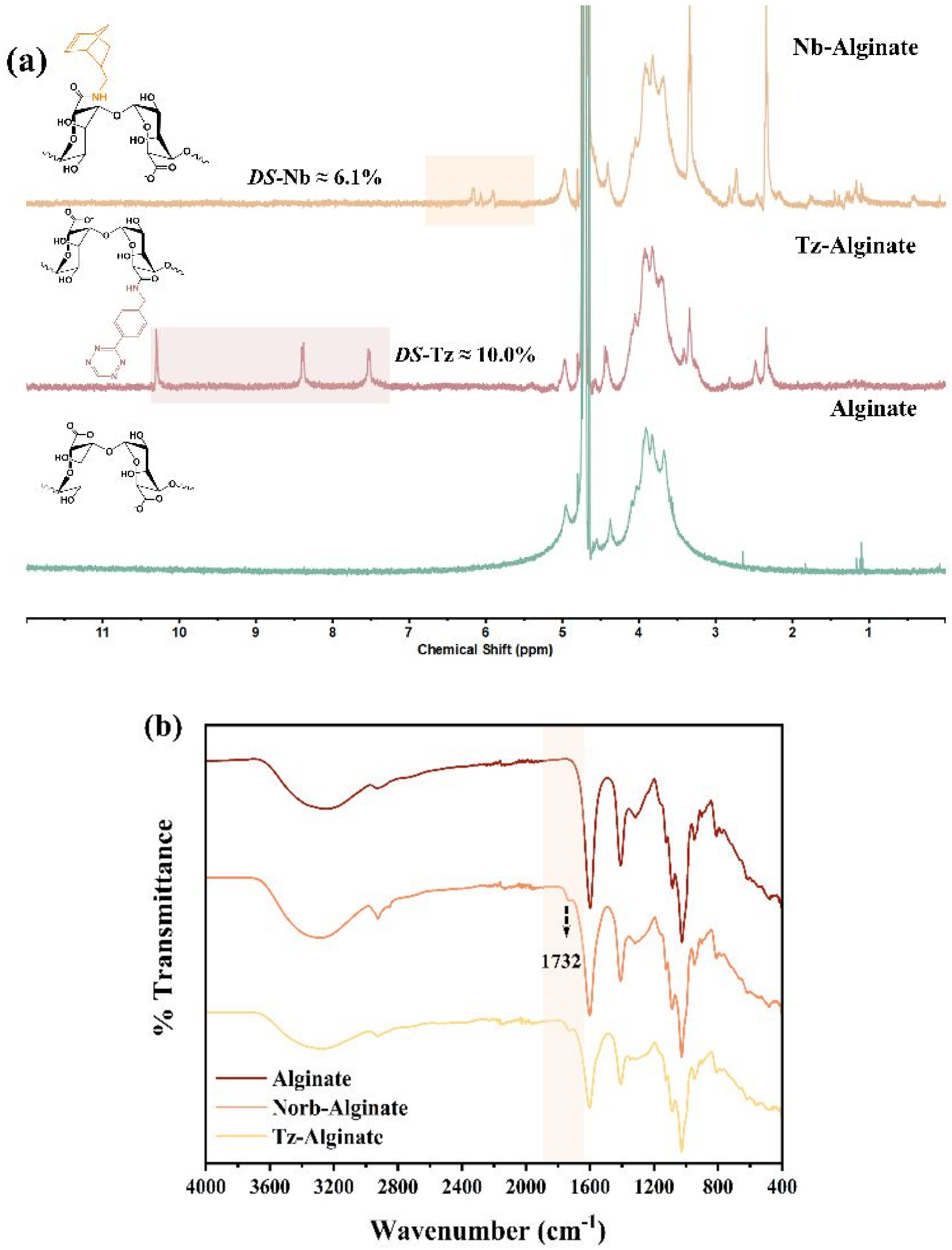

Supplementary Figure 1. Chemical characterization of functionalized alginates. (a) ^{1}H NMR spectra of alginate, Norb-functionalized alginate (Norb-Alginate), and Tz-functionalized alginate (Tz-Alginate). The characteristic vinyl proton peaks of Norb groups (δ = 5.92-6.18 ppm) and tetrazine proton peak (δ = 7.58-10.33 ppm) confirm successful functionalization. (b) FTIR spectra of alginate, Norb-Alginate, and Tz-Alginate showing the emergence of a new absorption band at ~1732 cm^{-1} corresponding to ester/amide linkages formed after modification.

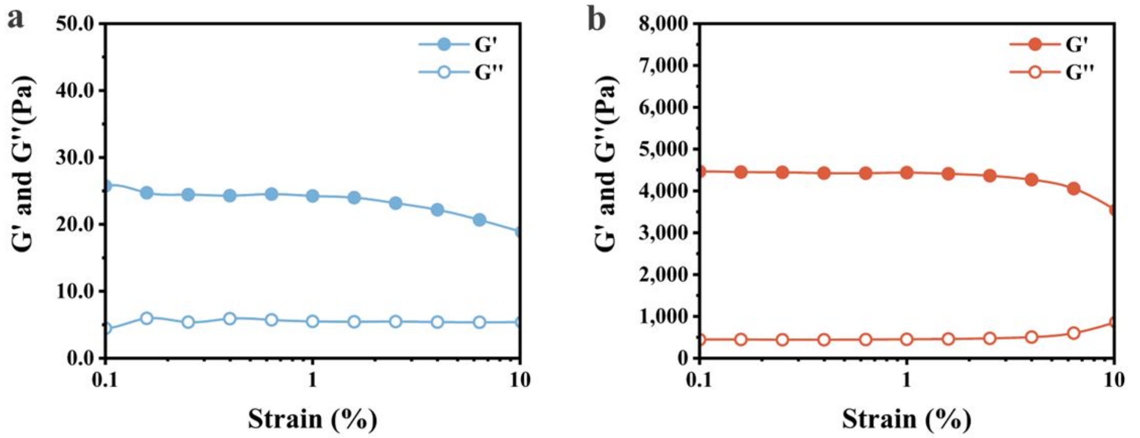

Supplementary Figure 2. Strain-sweep rheological analysis of collagen-alginate hybrid hydrogels (CAHs) treated with different Ca^{2+} concentrations: (a) 0 mM and (b) 50 mM.

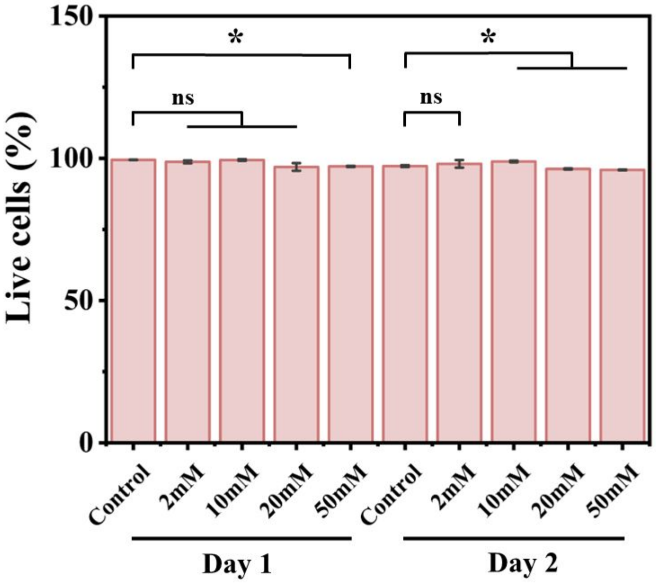

Supplementary Figure 3. Quantification of live ratios of NIH 3T3 fibroblasts cultured on CAHs after 1 and 4 days. Data are expressed as mean ± SD. Statistical significance: *$p < 0.05$, ns = not significant.

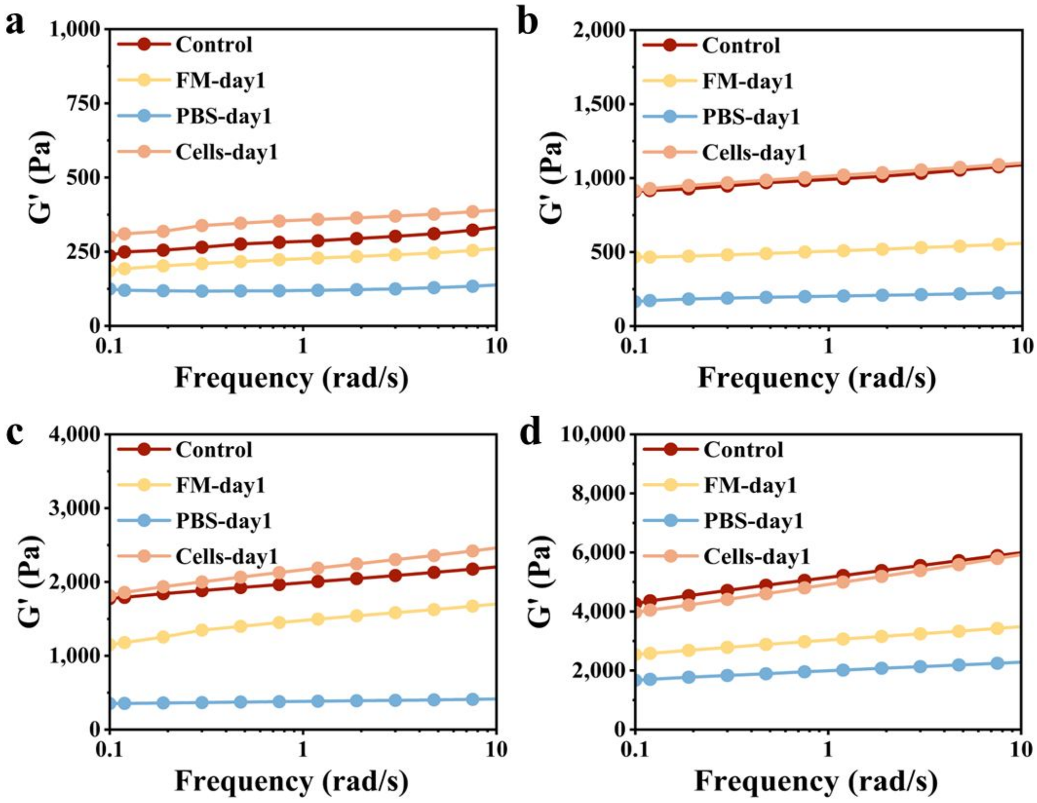

Supplementary Figure 4. Frequency sweep rheological analysis of CAHs cultured for 1 day under fibroblast medium (FM), phosphate-buffered saline (PBS), or FM supplemented with NIH 3T3 fibroblasts (Cells).

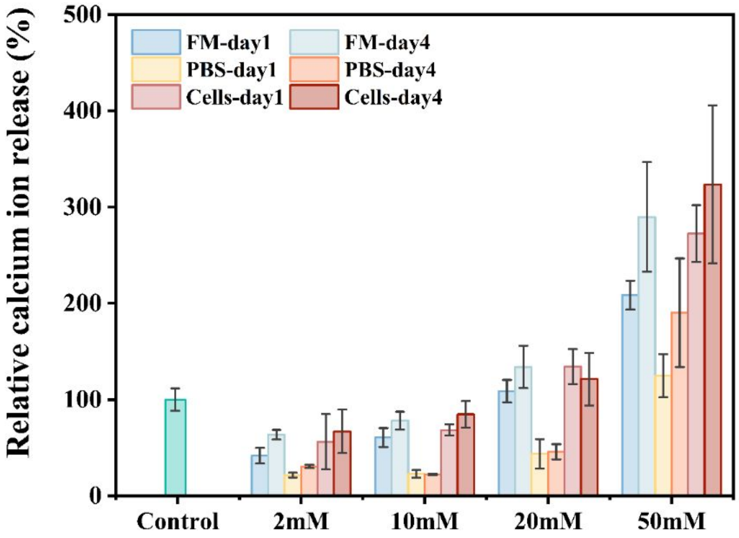

Supplementary Figure 5. Relative calcium ion release from CAHs crosslinked with different Ca^{2+} concentrations (2, 10, 20, and 50 mM) after incubation in fibroblast medium and PBS. Decellularized samples are denoted as FM, while cell-seeded samples are denoted as Cells. Calcium release was quantified after 1 and 4 days of culture, and values are expressed relative to the initial Ca^{2+} concentration of FM (3.50 mM). Error bars indicate standard deviations.

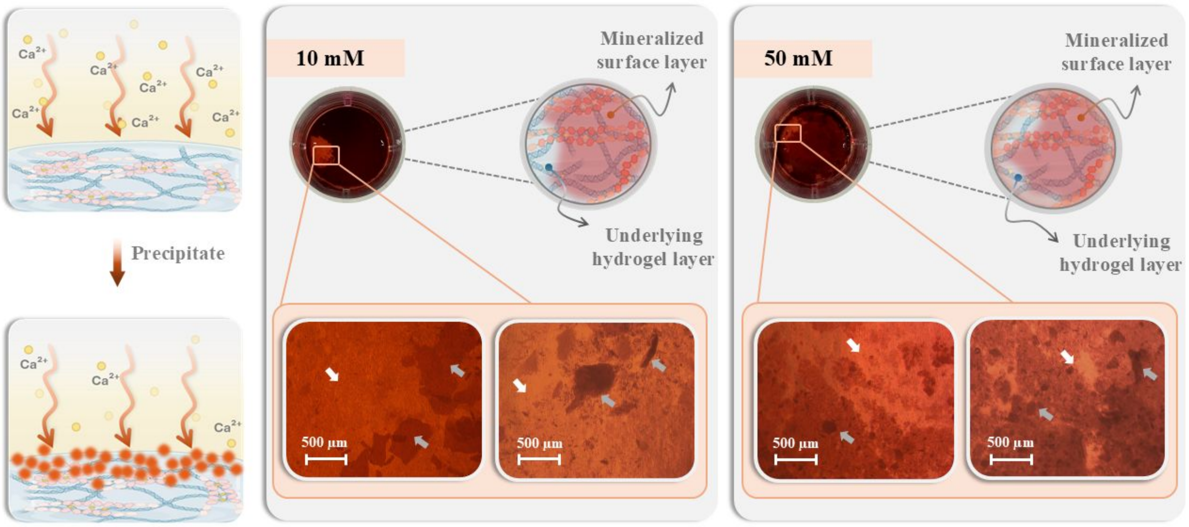

Supplementary Figure 6. Alizarin Red staining of surface mineralization in 10 and 50 mM CAHs. Schematic illustration (left) depicts Ca^{2+} diffusion and precipitation at the gel-medium interface. Representative macroscopic views and optical micrographs (5×) of 10 and 50 mM CAHs show Alizarin Red-positive mineralized surface layers (gray arrows) overlying the underlying hydrogel layer (white arrows). Scale bars = 500 µm.

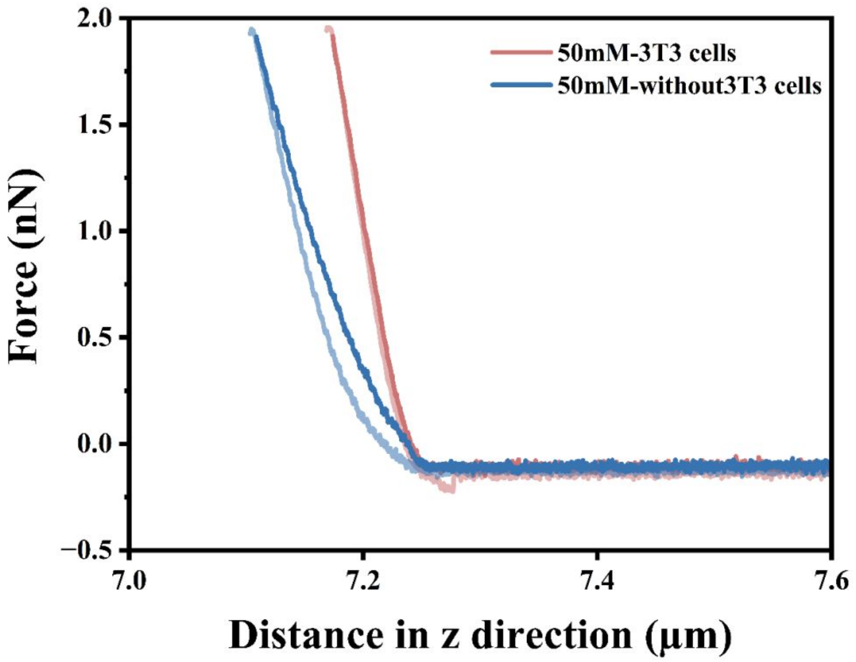

Supplementary Figure 7. Representative AFM force-displacement curves CAHs crosslinked with 50 mM Ca^{2+}, with or without NIH 3T3 fibroblasts. The bold curves correspond to the indentation process, reflecting the probe pressing into the hydrogel surface, while the lighter curves represent the retraction process, reflecting probe withdrawal from the hydrogel.
